# Blue-green endoscopy in a dog presenting chronic vomiting-regurgitation

**DOI:** 10.1186/s13620-015-0045-2

**Published:** 2015-07-30

**Authors:** Matteo Cerquetella, Andrea Spaterna, Beniamino Tesei, Gabrio Bassotti, Giacomo Rossi

**Affiliations:** School of Biosciences and Veterinary Medicine, University of Camerino, Via Circonvallazione, 93/95 – 62024 Matelica (MC), Italy; Gastroenterology, Hepatology and Digestive Endoscopy Section, Department of Medicine, University of Perugia Medical School, Perugia, Italy

**Keywords:** Blue, Green, Endoscopy, Dog, Esophagitis, Gastritis

## Abstract

A 2-year-old male Maremma sheepdog presenting with chronic vomiting-regurgitation was examined at the University Veterinary Teaching Hospital, Camerino University. An oesophagogastroscopy with a single blue + green (BG) filter restricting wavelengths from 400 to 550 nm was carried out. A conventional white light endoscopy showed a dilated oesophagus with mildly diffuse erythematous mucosa (more accentuated proximal to the cardia); some portions of the gastric mucosa were covered with fluids and appeared only slightly erythematous. A blue green endoscopy highlighted the oesophageal lesions in dark blue, which made them appear more clearly defined from the remaining mucosa. In the gastric antrum, a small, slightly darker blue roundish area was visible. This area did not show up under the white light endoscopy. A histopathological assessment of biopsy specimens from the distal oesophagus, antrum (including the area highlighted only by BG endoscopy) and gastric body showed chronic-active hyperplastic esophagitis and superficial squamous epithelial dysplasia, while gastric samples showed severe diffuse hyperaemic gastritis of the antrum and superficial diffuse atrophy of the gastric body. The authors believe that the use of a BG endoscopy could be useful in veterinary medicine to increase the diagnostic potential of endoscopic assessment in animals.

## Background

Narrow Band Imaging (NBI, Olympus) is an advanced technology frequently used in human medicine that allows endoscopic image enhancement [1]. This technique permits a better definition of the superficial mucosal structure and vascularization [2], especially when associated with high-definition and magnification techniques [3]. NBI is obtained by using filters on the white light source; it uses narrow wavelengths, like blue (400–430 nm) and green (525–555 nm) [4, 5], bandwidth 30 nm [2]; since a light with a short wavelength can only penetrate the mucosa superficially, it is possible to define its surface portions more clearly [4]. In addition, these wavelengths correspond to the range of haemoglobin absorption peak [3, 6].

There are few or no studies using endoscopic image enhancement in veterinary medicine. Here we report the endoscopic findings obtained using an endoscope equipped with a single blue-green filter (wavelengths from 400 to 550 nm), in a dog presenting with chronic vomiting-regurgitation.

## Case presentation

**Fig. 1:**
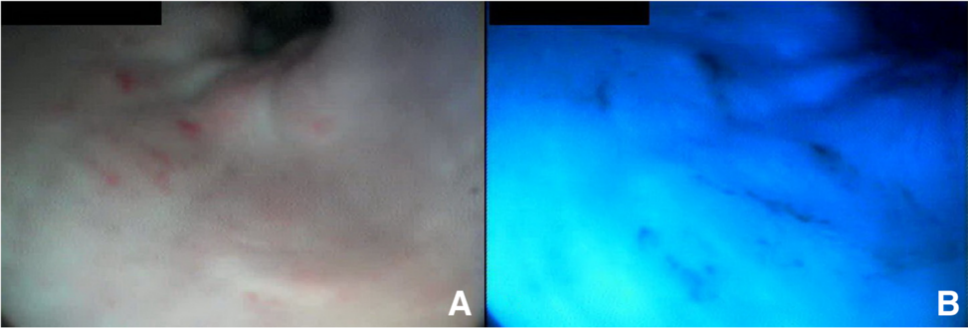
Distal oesophagus and cardia. **a** White light endoscopy. The oesophageal mucosa proximal to the cardia appears mildly erythematosus with small superficial areas of bleeding. **b** In this BG image blood and lesions are visible as dark blue areas. Lesions appear distributed in a manner comparable to that shown up by the white light endoscopy (Fig. 1a), but these areas appear more clearly defined compared to the remaining mucosa

**Fig. 2:**
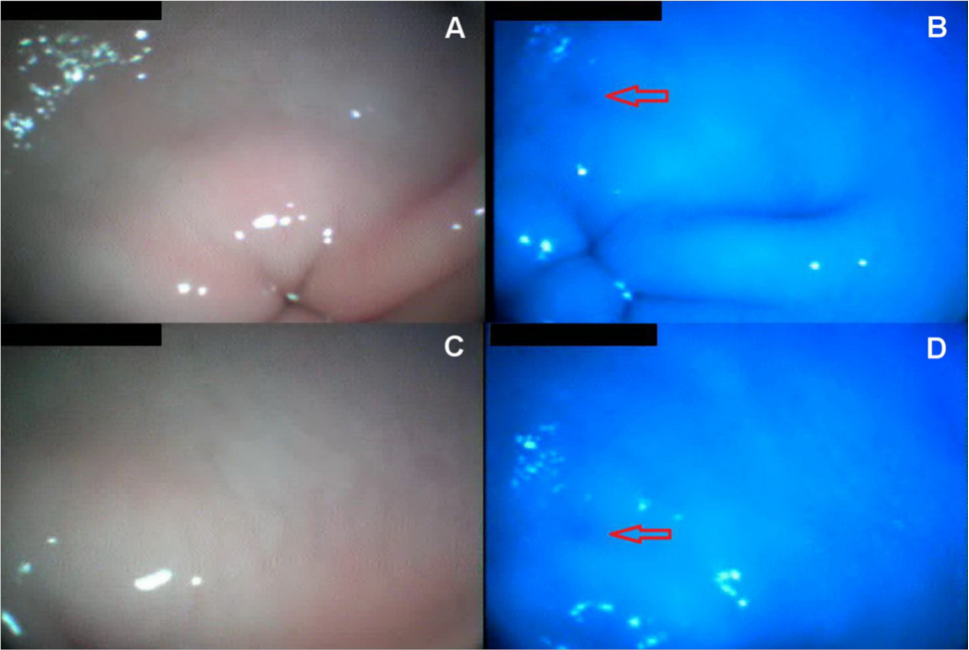
Antrum. **a** and **c** White light endoscopy. The mucosa of the antrum appear only slightly erythematous and no erosions are visible. **b** and **d** BG endoscopy shows a small round area (*red arrow*) that appears colored with a slightly darker blue tint. Pictures **c** and **d** show a more close-up view of the antrum

A 2-year-old male Maremma sheepdog presenting with chronic vomiting-regurgitation was examined at the University Veterinary Teaching Hospital, Camerino University. An oesophagogastroscopy under general anaesthesia (flexible video endoscope, 160 cm length and 9.8 cm Ø, Mercury Produzione®, Foligno, Italy) with a single blue + green (BG) filter, restricting wavelengths from 400 to 550 nm, was carried out. A conventional white light endoscopy showed a dilated oesophagus with mildly diffuse erythematous mucosa (more accentuated proximal to the cardia, where small superficial bleeding areas were also present; the mucosa reacted by bleeding at the slightest touch of the tip of the endoscope) (Fig. 1a). Within the stomach, some fluid was present on the gastric fundus and the visible gastric mucosa appeared slightly and diffusely erythematous. Using BG endoscopy, bleeding lesions of the distal oesophagus were visible as dark blue areas (Fig. 1b), more clearly defined from the remaining mucosa compared with a white light endoscopy (Fig. 1a). With regards to the antrum, no differences between the two imaging techniques were observed with respect to the appearance of the gastric mucosa, but BG imaging revealed a small roundish area of a slightly darker blue tint, not visible with the white light endoscopy (Fig. 2a, b, c and d). While waiting for the histopathology results, symptomatic therapy was administered. The histopathology of targeted BG biopsies from the distal oesophagus, the antrum (including the area highlighted only by the BG endoscopy) and the gastric body, showed chronic-active hyperplastic esophagitis with micro-erosions and moderate superficial squamous epithelial dysplasia, severe diffuse hyperaemic gastritis of the antrum and superficial diffuse atrophy of the gastric body; also GHLOs were present. After making a diagnosis of esophagitis and megaesophagus probably associated with gastroesophageal reflux disease (GERD) due to vomiting/gastritis, a percutaneous endoscopic gastrostomy (PEG) tube was inserted. It was then removed when the dog stopped vomiting and was slowly gaining weight.

In human medicine, a similar technology (NBI) using filters to narrow the light’s wavelength has shown interesting applications in oesophageal, as well as in gastric [7], small intestine (e.g. celiac disease) [5, 8], and colorectal diseases [7, 9], especially when associated with a magnification endoscopy [10].

## Conclusions

In this report, unlike white light endoscopy, BG endoscopy allowed us to obtain more clearly defined pictures of the oesophageal lesions distinguishing them from the remaining mucosa. With regard to the areas of the gastric body presenting histopathologically with  superficial atrophy, no differences were highlighted when visualized with a white light endoscopy and subsequently with a BG endoscopy. Affected areas were hardly detectable in the stomach with either technique, showing only mild and diffuse erythema. In the gastric antrum, however, a darker area was only visible by means of BG endoscopy (Fig. 2b and d), even though this finding was difficult to detect. The authors believe that further studies, like the present one, using endoscopes equipped with filters to restrict wavelengths are needed to understand the real potential of this technology in veterinary medicine. Nevertheless, the authors believe that the present study could represent a promising first step towards the understanding of possible applications of this technology in veterinary medicine, which could enhance our diagnostic tools when dealing with our animal patients.

## Abbreviation

NBI: Narrow Band Imaging
BG: Blue + green
